# Author Response to: “Randomized Controlled Trial of Simulation vs Standard Training for Teaching Medical Students High-quality Cardiopulmonary Resuscitation: The Methodological Issue”

**DOI:** 10.5811/westjem.2019.8.44853

**Published:** 2019-10-14

**Authors:** C. Eric McCoy

**Affiliations:** University of California, Irvine Medical Center, Department of Emergency Medicine, Orange, California

In reply:

Thank you for your interest in our study entitled “Randomized Controlled Trial of Simulation vs Standard Training for Teaching Medical Students High-quality Cardiopulmonary Resuscitation.” Your comments and questions were insightful and appreciated.

The participants in this study were all fourth-year medical students enrolled in a required emergency medicine (EM) clerkship. We excluded foreign medical students doing an observation rotation in the emergency department to evaluate a representative group of U.S. medical students. All students were in their final year in medical school, on a required EM rotation, and had previous simulation experience with simulation as part of their medical school curriculum. The participants were balanced with regard to these independent variables.

We chose a prospective, randomized controlled trial study design as this is the optimal methodological approach to evaluate the effectiveness of an intervention compared to a control. Randomization affords the generation of two prognostically balanced groups such that any difference observed at the end of the trial can be attributed to the intervention. Furthermore, randomization is the optimal methodological approach to control for both known and unknown confounders.

The Kruskal-Wallis test is an analytical approach that allows for the assessment of significant differences on a continuous dependent variable by a categorical independent variable (with two or more groups). Since it is a non-parametric method, this test does not assume a normal distribution of the data. The Kruskal-Wallis test can be used for both continuous and ordinal-level dependent variables and is used for comparing two or more independent samples of equal or different sample sizes. It extends the Mann-Whitney U test, which is used for comparing only two groups.

Our decision to evaluate the effect size of a 5-millimeter difference in compression depth between the two groups was a balance between identifying a clinically relevant difference within the practical context of a study protocol with the power to detect that difference with statistical significance. To our knowledge, there are no studies to date evaluating a difference in compression depth smaller than that reported in our trial.

A confounder is an underlying variable that is both linked to the exposure of interest and independently associated with the outcome under study. One of the major benefits of randomization is that this is the optimal methodological approach to control for both known and unknown confounding variables. We chose to conduct a prospective randomized controlled trial for this reason, as this is the gold standard when evaluating for and establishing a causal relationship between independent and dependent variables.

Our methods for data collection can be found in the methods and measurements section. In short, the performance metrics measured for high-quality CPR in our study were specifically defined in the AHA guidelines. The high-fidelity simulation software we used allows for the real-tine collection of chest compression rate, depth and recoil. Video capture of each scenario was performed with B-Line Medical SimBridge software (Washington, DC). Data input was done via standardized abstractions sheets.

Thank you again for your insightful questions, comments, and interest in our study.

